# Crystal structure and initial characterization of a novel archaeal-like Holliday junction-resolving enzyme from *Thermus thermophilus* phage Tth15-6

**DOI:** 10.1107/S2059798321012298

**Published:** 2022-01-24

**Authors:** Josefin Ahlqvist, Javier A. Linares-Pastén, Maria Håkansson, Andrius Jasilionis, Karolina Kwiatkowska-Semrau, Ólafur H. Friðjónsson, Anna-Karina Kaczorowska, Slawomir Dabrowski, Arnþór Ævarsson, Guðmundur Ó. Hreggviðsson, Salam Al-Karadaghi, Tadeusz Kaczorowski, Eva Nordberg Karlsson

**Affiliations:** a Biotechnology, Department of Chemistry, Lund University, PO Box 124, 221 00 Lund, Sweden; b SARomics Biostructures, Medicon Village, 223 81 Lund, Sweden; c Laboratory of Extremophiles Biology, Department of Microbiology, Faculty of Biology, University of Gdansk, ul. Wita Stwosza 59, 80-308 Gdansk, Poland; d Matís, Vínlandsleið 12, 113 Reykjavík, Iceland; e Collection of Plasmids and Microorganisms, University of Gdansk, ul. Wita Stwosza 59, Gdansk 80-308, Poland; f A&A Biotechnology, al. Zwycięstwa 96/98, 81-451 Gdynia, Poland; g Department of Biology, School of Engineering and Natural Sciences, University of Iceland, Sturlugata 7, IS-102 Reykjavik, Iceland

**Keywords:** archaeal-like Holliday junction-resolving enzymes, structure–function relationship, signature motifs, thermophilic bacteriophages

## Abstract

This study describes the production, characterization and structure determination of a novel Holliday junction-resolving enzyme. The enzyme, termed Hjc_15-6, is encoded in the genome of phage Tth15-6, which infects *Thermus thermophilus*. Hjc_15-6 was heterologously produced in *Escherichia coli* and high yields of soluble and biologically active recombinant enzyme were obtained in both complex and defined media. Amino-acid sequence and structure comparison suggested that the enzyme belongs to a group of enzymes classified as archaeal Holliday junction-resolving enzymes, which are typically divalent metal ion-binding dimers that are able to cleave X-shaped dsDNA–Holliday junctions (Hjs). The crystal structure of Hjc_15-6 was determined to 2.5 Å resolution using the selenomethionine single-wavelength anomalous dispersion method. To our knowledge, this is the first crystal structure of an Hj-resolving enzyme originating from a bacteriophage that can be classified as an archaeal type of Hj-resolving enzyme. As such, it represents a new fold for Hj-resolving enzymes from phages. Characterization of the structure of Hjc_15-6 suggests that it may form a dimer, or even a homodimer of dimers, and activity studies show endonuclease activity towards Hjs. Furthermore, based on sequence analysis it is proposed that Hjc_15-6 has a three-part catalytic motif corresponding to E–SD–EVK, and this motif may be common among other Hj-resolving enzymes originating from thermophilic bacteriophages.

## Abstract 2

The first structure of a novel archaeal-like Holliday junction-resolving enzyme originating from a thermophilic phage was determined and its function in cleaving X-shaped dsDNA was demonstrated. Furthermore, a novel signature motif for Holliday junction-resolving enzymes originating from thermophilic bacteriophages is proposed.

## 1. Introduction

Holliday junction-resolving enzymes are nucleases that cleave four-way DNA–Holliday junctions (Hjs) into two unconnected DNA duplexes. Hjs are common intermediates during meiotic and mitotic genetic recombination, and Hj-resolving enzymes have been isolated from all types of eukaryotic and prokaryotic cells and their viruses (Wyatt & West, 2014 ▸). Hjs were first presented by Robin Holliday in 1964, when he suggested a model for gene conversion during meiosis in fungi (Holliday, 1964 ▸), claiming that two homologous chromosomes paired between complementary sequences lead to the formation of a cross-stranded structure that physically links the two component helices. Since then, other models of how and why two dsDNA helices may cross-link have been reported. This type of four-way DNA structure at the point of strand exchange has become known as an Hj, and the enzymes that resolve them are referred to as Hj-resolving enzymes.

Apart from playing a major role in gene conversion during meiosis, it has been suggested that Hjs and Hj-resolving enzymes are involved in processes such as DNA repair and the introduction of plasmid and phage DNA into host genomes (Wyatt & West, 2014 ▸), and Hj-resolving enzymes are now considered to be key enzymes in DNA recombination (Aravind *et al.*, 2000 ▸). For instance, the 157-residue endo VII encoded by gene 49 of bacteriophage T4 resolves branched multimeric T4 DNA before packaging it into phage heads (Biertümpfel *et al.*, 2007 ▸; Kemper & Brown, 1976 ▸; Kemper & Janz, 1976 ▸; Mizuuchi *et al.*, 1982 ▸). In 2001, Birkenbihl and coworkers presented the characterization, including the isolation and purification, of two similar Hj-cleaving enzymes from two different viruses, SIRV1 and SIRV2, that infect the archaeon *Sulfolobus islandicus* (Birkenbihl *et al.*, 2001 ▸). At the time, the phage enzymes endonuclease VII from phage T4 and endonuclease I from phage T7 (T7 endo I), the bacterial proteins RuvC and RusA from *Escherichia coli* and the yeast enzymes CCE1 from *Saccharomyces cerevisiae* and YPD2 from *Schizosaccharomyces pombe* 2.3 were the best-studied members of the growing group of structure-specific endo­nucleases. While the latter two enzymes are homologous, no significant sequence similarity exists between the other proteins.

Hj-resolving enzymes are diverse both in sequence and in structural organization (Lilley & White, 2000 ▸). However, this may not be surprising considering the different evolutionary origins, purposes and outcomes of the creation and resolution of Hjs. Aravind and coworkers analysed the structural and evolutionary relationships of various Hj-resolving enzymes and related nucleases, suggesting that an Hj-resolving enzyme function has evolved independently from at least four distinct structural folds (RNase H, endonuclease, endo­nuclease VII–colicin E and RusA; Aravind *et al.*, 2000 ▸). From this work, the endonuclease fold (the structural prototype of which is the phage λ exonuclease) was shown to encompass a far greater diversity of nucleases than previously suspected, including archaeal Hj-resolving enzymes, repair nucleases such as the RecB and Vsr enzymes, and a variety of predicted nucleases. The authors state that the structural prototype for archaeal Hj-resolving enzymes originates from *Pyrococcus furiosus*, and was isolated, cloned and characterized by Komori *et al.* (1999 ▸).

Even though the origin, amino-acid sequence, fold and overall biological functionality of Hj-resolving enzymes vary, these nucleases often share a number of characteristics (Lilley, 2017 ▸). For instance, they most often have a high proportion of positively charged amino acids, enabling them to bind DNA with high affinity. Analyses of the three-dimensional structures of Hj-resolving enzymes have shown that their active sites generally contain three or four acidic residues that are required for metal binding and catalysis. In addition, a divalent metal ion, usually Mg^2+^ or Mn^2+^, that is essential for DNA cleavage but not for DNA binding is present. Furthermore, Hj-resolving enzymes have been reported to be dimeric, allowing the use of twin active sites to catalyse two coordinated incisions (Wyatt & West, 2014 ▸).

Several non-archaeal-like Hj-resolving enzymes from viruses have had their structures determined and/or their activities confirmed following endonuclease VII from phage T4 (Kemper & Brown, 1976 ▸; Kemper & Janz, 1976 ▸; Raaijmakers *et al.*, 1999 ▸; Ariyoshi *et al.*, 1994 ▸; Mizuuchi *et al.*, 1982 ▸), which was the first enzyme known to resolve an Hj. In recent years, structures of fowlpox Hj-resolving enzyme (Culyba *et al.*, 2009 ▸; Li *et al.*, 2020 ▸) and canarypox Hj-resolving enzyme (Li *et al.*, 2016 ▸), for example, have been determined.

In this study, carried out within the frame of the VIRUS-X project (Aevarsson *et al.*, 2021 ▸), a novel archaeal-like Hj-resolving enzyme, Hjc_15-6, originating from phage Tth15-6 is described. Phage Tth15-6, which infects the thermophilic bacterium *Thermus thermophilus*, was originally isolated from a coastal hot spring at Reykjanes in Isafjardardjup, Iceland (G. Ó. Hreggviðsson, personal communication). Tth15-6 belongs to the long-tailed phage morphotype of the *Siphoviridae* family in the *Caudovirales* order (Yu *et al.*, 2006 ▸). *T. thermophilus* is a Gram-negative, thermophilic heterotrophic bacterium that is found in coastal hot springs all around the world. The genomes of other viruses infecting *T. thermophilus* strains have been sequenced, for example *Thermus* phage φYS40 (Naryshkina *et al.*, 2006 ▸), *Thermus* virus IN93 (Matsushita & Yanase, 2008 ▸), *Thermus* phage φTMA (Tamakoshi *et al.*, 2011 ▸), *Thermus* virus P23-77 (Jalasvuori *et al.*, 2009 ▸), *Thermus* phage TSP4 (Lin *et al.*, 2010 ▸), *Thermus* phage G20c (Xu *et al.*, 2017 ▸), *Thermus* phage P23-45 and *Thermus* phage P74-26 (Minakhin *et al.*, 2008 ▸), but only a few enzymes have so far been characterized from these bacteriophages.

The study of Hjc_15-6 reported here encompasses structural analysis, including the crystal structure, and some studies of the biological function of this putative Hj-resolving enzyme. Furthermore, the sequence relationship between this enzyme and related Hj-resolving enzymes of viral and bacterial origin, including the putative enzymes encoded in the deposited genomes of *Thermus* phage P23-45 and *Thermus* phage P74-26, is investigated. This paper describes, to our knowledge, the first crystallized Hj-resolving enzyme from a thermo­philic phage that may be defined as an archaeal type both from a sequence and structural perspective. Therefore, it also presents a new fold among Hj-resolving enzymes originating from phages.

## 2. Materials and methods

### 2.1. Isolation of phage Tth15-6

An overnight culture of *T. thermophilus* MAT15 was mixed with 4 ml of water from a coastal hot spring at Reykjanes in Isafjardardjup, Iceland. The sampled water had been filtered through a 0.22 µm pore-size filter prior to mixing with the overnight culture. The obtained suspension was incubated at 65°C for 30 min. 5 ml soft agar with 10 m*M* MgCl_2_ was then added and poured onto Medium 166 solidified with agar and the plates were incubated at 65°C overnight (Hjorleifsdottir *et al.*, 2001 ▸). Subsequently, plaques were aseptically transferred with a Pasteur pipette into microcentrifuge tubes with 100 µl 10 m*M* MgCl_2_ and stored at 4°C. The plaque solution was then diluted 10^3^–10^4^ with 10 m*M* MgCl_2_, and 100 µl of the diluted phage solution was added to 900 µl of an overnight culture of *T. thermophilus* MAT15 and incubated at 65°C for 30 min. Soft agar was then added and the mixture was poured onto plates with Medium 166. After overnight growth, the plaques were picked and the whole procedure was repeated four times to purify the phage particles.

### 2.2. DNA isolation, sequencing and identification of the putative DNA-resolving enzyme gene

Phage-amplification enrichment for DNA isolation was achieved using a 10^3^–10^4^ phage dilution, which gave confluent lysis on soft agar plates. The soft agar was scraped from 20 plates and suspended in 100 ml 10 m*M* MgCl_2_. The mixture was incubated at room temperature and 600 rev min^−1^ for 2 h and was then centrifuged at 11 000*g* for 20 min. The supernatant was filtered through a sterile 0.22 µm pore-size filter and the titre was analysed. Phage DNA was isolated from the supernatant with a titre of >10^9^ pfu ml^−1^ using the NucleoSpin Plasmid kit (Macherey-Nagel), performing binding in NT2 buffer. A sequencing library was constructed using the Nextera XT method (Illumina) and sequenced on an Illumina MiSeq System (Illumina) sequencing platform using the Miseq Reagent Kit v3 and 2 × 300 bp sequencing cycles. Sequences were trimmed for quality using *Trimmomatic* version 0.36 and assembled using the *SPAdes* version 3.12.0 assembly algorithm to produce a linear consensus sequence of 76 134 bp.

Analysis of the predicted ORF in the sequenced genome of phage Tth15-6 was performed using the *RAST* server (https://rast.nmpdr.org/; Aziz *et al.*, 2008 ▸). Genome annotation suggested the presence of a gene encoding a putative DNA-resolving enzyme/helicase-like enzyme. Gene-sequence annotation allowed the design of primers for PCR amplification of the gene from the isolated genomic DNA.

### 2.3. Gene cloning

The gene encoding Hjc_15-6 was amplified from the phage Tth15-6 genomic DNA by PCR with forward primer 5′-TCTCATATGTCTAAAGATAAAGGA-3′ (the NdeI restriction site is underlined) and reverse primer 5′-TAGCTCCTCGAGACCTGTGAAGTC-3′ (the XhoI restriction site is underlined). The amplified gene was inserted into the pET-21b(+) vector (Novagen), giving the construct pET-21b::Hjc_15-6. A PCR reaction mixture with a final volume of 20 µl was prepared with 10 µl Master Mix iProof (2×) (Bio-Rad), 1 ng DNA template, 0.5 µ*M* of each primer, 3%(*v*/*v*) DMSO and nuclease-free water. The reaction was preheated at 98°C for 120 s and 29 cycles of amplification were then applied with denaturation at 98°C for 20 s, annealing at 65°C for 30 s and elongation at 72°C for 15 s, with a final elongation at 72°C for 420 s. The PCR products were purified with the QIAquick PCR purification kit (Qiagen). Both the amplicon and the pET-21b(+) vector were digested with the NdeI and XhoI restriction endonucleases. The digestion products were purified from the agarose gel with a QIAquick Gel Extraction kit (Qiagen) and ligated with T4 ligase according to the manufacturer’s instructions. *E. coli* DH5α (Novagen) cells were transformed with the ligation products. Colonies harbouring the construct pET-21b::Hjc_15-6 were determined by colony PCR. The expression-construct sequence was verified by sequencing, confirming that the gene was cloned in frame with the C-terminal hexahistidine tag encoded by the expression vector. The gene was deposited in the NCBI GenBank with accession number MW788030.

### 2.4. Protein production

The gene encoding Hjc_15-6 with an attached C-terminal hexahistidine tag was initially expressed in *E. coli* BL21(DE3) cells (Novagen) using lysogeny broth (LB) for cultivation in shake flasks and was subsequently upscaled to 2.5 l in a 3 l fermenter using mAT medium.

The cultivation in shake flasks was performed in baffled Erlenmeyer flasks filled to not more than a quarter of the flask volume with LB (10 g l^−1^ tryptone, 5 g l^−1^ yeast extract, 5 g l^−1^ NaCl) supplemented with 100 µg ml^−1^ ampicillin. Expression cultures were inoculated to 0.5%(*v*/*v*) with overnight culture and were cultivated at 37°C and 200 rev min^−1^. Heterologous overexpression of Hjc_15-6 was induced with 1 m*M* isopropyl β-d-1-thiogalactopyranoside (IPTG) when the culture reached an OD_620 nm_ of 0.7–0.9. Induction was performed for 4 h at 37°C and 200 rev min^−1^.

Target enzyme production was upscaled to 2.5 l in a 3 l fermenter (Belach Bioteknik) using defined mAT medium [2 g l^−1^ ammonium sulfate, 14.6 g l^−1^ K_2_HPO_4_, 3.2 g l^−1^ NaH_2_PO_4_·H_2_O and 0.5 g l^−1^ ammonium hydrocitrate supplemented with 2 ml l^−1^ 1 *M* MgSO_4_ and 2 ml l^−1^ trace-elements solution (Holme *et al.*, 1970 ▸) as well as 20 ml l^−1^ 50%(*w*/*v*) d-glucose (de Maré *et al.*, 2005 ▸)] supplemented with 100 µg ml^−1^ ampicillin. Cultivation in the fermenter was performed at a constant pH of 7.0 at 37°C with at least 20% dissolved oxygen tension (DOT). The aeration was set to a constant 1 v.v.m. (volume of air per volume of liquid per minute). The pH was maintained by titration with 12.5%(*w*/*v*) ammonia solution, while the DOT was controlled by the stirrer speed (initial speed 300 rev min^−1^). The fermenter was inoculated to 4%(*v*/*v*) with overnight culture cultivated using mAT medium at 30°C and 200 rev min^−1^. Heterologous overexpression of Hjc_15-6 was induced with 1 m*M* IPTG when the culture in the fermenter reached an OD_620 nm_ of 3. Induction was performed for 1 h at 37°C.

After termination of cultivation, harvesting was performed by centrifugation of the chilled cultures at 3800*g* for 10 min at 4°C. The collected cell pellets were stored frozen before purification of Hjc_15-6.

### 2.5. Selenium derivatization of Hjc_15-6

Hjc_15-6 was derivatized with selenium by seleno-l-methionine (SeMet) incorporation, producing recombinant protein in the methionine-auxotrophic *E. coli* strain B834(DE3) (Novagen) cells using mAT medium supplemented with 50 mg l^−1^ SeMet. Cultivation in shake flasks was performed for derivatization (Turner *et al.*, 2007 ▸). Expression cultures were inoculated to 1%(*v*/*v*) with cell suspension prepared by washing fresh overnight culture from LB agar plates with mAT medium twice. Induction, harvesting and cell-pellet storage were performed as described previously.

### 2.6. Protein purification

The collected cell pellets were thawed on ice and resuspended in binding buffer (100 m*M* Tris–HCl pH 7.4, 500 m*M* NaCl). The cells were disrupted by sonication (20 cycles of 30 s on, 30 s off with 60% amplitude) using an UP400s sonicator (Hielscher Ultrasound Technology) in an ice bath. The soluble protein fraction was separated from the cell debris by centrifugation at 17 000*g* for 20 min at 4°C. Hjc_15-6 was purified by nickel-affinity chromatography using a 5 ml 16 × 25 mm HisTrap HP column (GE Healthcare Life Sciences). Elution was performed with a linear 8 ml gradient to 100% elution buffer (100 m*M* Tris–HCl pH 7.4, 500 m*M* NaCl, 500 m*M* imidazole). Fractions containing Hjc_15-6 were combined and dialyzed (1:5000) for 12–16 h at 4°C. The dialyzed protein sample was subjected to crystallization trials. The purity of Hjc_15-6 was polished by combining nickel affinity with Heparin Sepharose chromatography for use in activity studies. Hjc_15-6 was purified by Heparin Sepharose chromatography using a 1 ml 7 × 25 mm HiTrap Heparin HP column (GE Healthcare Life Sciences). Elution was performed with a linear salt gradient. Desalting was performed by dialysis. The purity and integrity of Hjc_15-6 were assessed by glycine SDS–PAGE using 4–20% gradient gels. The protein concentration was estimated by measuring *A*_280 nm_ with a NanoDrop 1000 (Thermo Fisher Scientific).

### 2.7. Mass spectrometry

Purified samples of Hjc_15-6, produced using complex (LB) medium or defined (mAT) medium as described previously, were analysed by mass spectrometry (MS). The protein sample concentration was adjusted to 5 mg ml^−1^ and it was frozen in liquid nitrogen and stored frozen at −80°C prior to MS analysis. As a precaution, the samples were spun down after thawing on ice.

MS spectra were acquired using an Autoflex Speed MALDI–TOF/TOF mass spectrometer (Bruker Daltonics) in positive linear mode. 0.5 µl matrix solution consisting of 5 mg ml^−1^ α-cyano-4-hydroxycinnamic acid, 80%(*v*/*v*) aceto­nitrile, 0.1%(*w*/*v*) trifluoroacetic acid (TFA) was added to 1 µl Hjc_15-6 sample on a MALDI stainless-steel plate. A total of 5000 laser shots were collected per spectrum and were calibrated using the Protein I calibration standard (Bruker Daltonics) containing six internal standard proteins (insulin, *m*/*z* 5734.52; cytochrome *c*, *m*/*z* 6181.05; myoglobin, *m*/*z* 8476.66; ubiquitin I, *m*/*z* 8565.76; cytochrome *c*, *m*/*z* 12 360.97; myoglobin, *m*/*z* 16 952.31).

### 2.8. Dynamic light scattering

Purified samples of Hjc_15-6 produced in complex (LB) medium or defined (mAT) medium as described previously were analysed by dynamic light scattering (DLS) at a 173° backscattering angle at 25°C using a Zetasizer Nano ZS instrument (Malvern Panalytical). The concentration of the protein sample after filtration through a 0.22 µm pore-size filter was adjusted to 0.25 mg ml^−1^ with SPG (succinic acid–NaH_2_PO_4_–glycine) buffer pH 7.0. The purified protein from defined (mAT) and complex (LB) medium had an initial concentration of 5.4 and 11.4 mg ml^−1^, respectively (from the *A*_280 nm_ measured using a NanoDrop). The purified protein produced in defined medium had been stored for six months at −20°C, while the protein produced in complex medium was freshly made. After concentration adjustment, the protein samples were centrifugated at 14 000*g* for 5 min. DLS measurements were performed twice in triplicate at 20°C using ZEN0040 micro-cuvettes (Malvern Panalytical). In addition, the 50 µl samples were incubated at 25°C for 1 min before measurement. The size of the protein particles was calculated from the average size distribution obtained from six measurements: two measurements in triplicate.

### 2.9. Screening for resolving activity

The resolving activity of Hjc_15-6 was studied using fluorescent X-shaped DNA as a Holliday junction substrate. The substrate was assembled from DNA oligomers 1–4, with fluorescent-tagged CGA triplets at the 5′ position, as detailed in Table 1 ▸.

100 m*M* oligo DNA was mixed to give a concentration of 10 m*M* DNA. The solution was heated for 3 min at 94°C and then cooled for hybridization. 1 µl of 10 m*M* hybridized DNA containing approximately 10 pmol X-shaped DNA was used in a reaction mixture with 1 µl 1.3 mg ml^−1^ purified Hjc_15-6 (∼35 pmol) that had been produced in complex medium and purified once or twice as described above. The final buffer concentration in the reaction mixture was 10 m*M* Tris–HCl pH 8.5, 100 m*M* KCl, 10 m*M* MgCl_2_ supplemented, when necessary, with 5 m*M* ATP. The reaction mixtures were incubated at 50°C for 30 min. The result of the reactions was visualized on an 8% TBE polyacrylamide gel (Sambrook *et al.*, 1989 ▸). As a control for the digestion of nonbranched dsDNA, 1 µg blunt-end double-stranded phage λ DNA (a polymerase-treated phage λ DNA from A&A Biotechnology, catalogue No. 3500-500, DNA marker λ/HindIII) was used as a substrate DNA (instead of X-shaped DNA) with Hjc_15-6 that had been purified twice. The reaction mixture and incubation time were as before, although the incubation temperature was kept at 37°C. The result was visualized on a 1% agarose gel (Sambrook *et al.*, 1989 ▸).

### 2.10. Crystallization and X-ray diffraction data collection

Native crystals were obtained using Hjc_15-6 produced in defined (mAT) medium and purified as described previously. The crystals were grown using protein solution consisting of 5.4 mg ml^−1^ Hjc_15-6 in 20 m*M* SPG buffer system pH 7.0 (Molecular Dimensions). The crystallization drops were set up using 200 nl protein solution, 50 nl seed solution and 150 nl reservoir solution (1.8 *M* ammonium sulfate, 0.1 *M* sodium acetate pH 4.2). The seed solution (crushed native Hjc_15-6 crystals in 100 m*M* sodium acetate pH 4.8, 2 *M* ammonium sulfate, 0.5 mg ml^−1^ native Hjc_15-6) was obtained using seed beads (Hampton Research) from initial native crystals grown under the same conditions. The crystals were grown at 20°C in MRC 3-well plates over 40 µl reservoir solution. Since attempts to use the molecular-replacement method to determine the structure did not succeed, selenium-derivatized (SeMet) Hjc_15-6 was produced. One SeMet Hjc_15-6 crystal was grown in the same way as described for the native Hjc_15-6 crystals but using protein at 3 mg ml^−1^. Another SeMet Hjc_15-6 crystal was grown without seeding in a 200 nl protein drop with 200 nl reservoir solution (1.8 *M* ammonium sulfate, 0.1 *M* sodium acetate pH 4.2) added. All crystals appeared in 1–2 weeks. Prior to data collection and just before flash-cooling in liquid nitrogen, the crystals were briefly transferred to a cryosolution [100 m*M* sodium acetate pH 4.2, 1.8 *M* ammonium sulfate, 25%(*v*/*v*) glycerol].

Ten data sets for native Hjc_15-6 were collected to 2.55 Å resolution at a wavelength of 1.7701 Å on beamline I04 at Diamond Light Source (DLS), United Kingdom. The data were processed in *XDS* (Kabsch, 2010 ▸) and all data sets were scaled using *XSCALE* (Kabsch, 2010 ▸). These data were collected in an attempt to obtain sulfur SAD (single anomalous determination) phases, but turned out to give highly redundant and good native data, although the sulfur signal was too weak for phasing using the *CRANK*2 pipeline (Skubák *et al.*, 2018 ▸). Instead, data sets for SeMet Hjc_15-6 were collected on beamline I04 at DLS at the peak wavelength for selenium (0.9795 Å). The data sets were processed in *XDS* (Kabsch, 2010 ▸) and two of these data sets were combined using *XSCALE* (Kabsch, 2010 ▸) to 2.50 Å resolution. Significant anomalous signal extended to 3.2 Å resolution. The structure was determined using the *CRANK*2 pipeline (Skubák *et al.*, 2018 ▸) included in the *CCP*4 suite using the combined peak data sets and the Hjc_15-6 sequence. Subsequent model building was performed in *Coot* (Emsley *et al.*, 2010 ▸) with refinement in *REFMAC*5 (Murshudov *et al.*, 2011 ▸) followed by refinement in *BUSTER* (Bricogne *et al.*, 2011 ▸).

### 2.11. Bioinformatic tools and software

The pI value of Hjc_15-6 was estimated as an average value from the *IPC* isoelectric point calculator (http://isoelectric.org/; Kozlowski, 2016 ▸) and the *ProtParam* tool available via the Expasy portal (https://web.expasy.org/protparam/; Gasteiger *et al.*, 2005 ▸). A theoretical molecular-mass calculation was also performed using *ProtParam*. A sequence-similarity search was performed using *BlastP* (https://blast.ncbi.nlm.nih.gov/Blast.cgi; NCBI Resource Coordinators, 2016 ▸) against the nonredundant protein-sequence database (in September 2021), excluding models (XM/XP), nonredundant RefSeq proteins (WP) and uncultured/environmental sample sequences, but otherwise using the default settings. Evolutionary analysis was performed and a phylogenetic tree was obtained with *MEGA* version *X* (Kumar *et al.*, 2018 ▸) using the data from the *BlastP* search. Alignments were performed using *Clustal Omega* (https://www.ebi.ac.uk/Tools/msa/clustalo/) through *Jalview* version 2.11.14 (Waterhouse *et al.*, 2009 ▸) using the *ClustalO* web service with the default settings, unless stated otherwise. Protein attribution to (super)families was performed with *InterPro* version 83.0 (http://www.ebi.ac.uk/interpro/; Blum *et al.*, 2021 ▸). Conservative sequence motifs were investigated in the NLM Conserved Domain Database (CDD) version 3.18 (Lu *et al.*, 2020 ▸). Illustrations of protein structures were prepared with *CCP*4*MG* (McNicholas *et al.*, 2011 ▸) and *UCSF Chimera* version 1.15 (Pettersen *et al.*, 2004 ▸). Protein structure comparisons were made with the PDB coordinates for chain *A* of Hjc_15-6 using the *DALI* server (http://ekhidna2.biocenter.helsinki.fi/dali/; Holm, 2020 ▸). All protein illustrations and protein structure comparisons were made using the SeMet-derivatized model of Hjc_15-6 (PDB entry 7bgs), unless stated otherwise. The predicted oligomeric state of Hjc_15-6 was analysed with the coordinates of the native and SeMet-derivatized structures using the *Protein Interfaces, Surfaces and Assemblies* service (*PISA*) at the European Bioinformatics Institute (http://www.ebi.ac.uk/pdbe/prot_int/pistart.html; Krissinel & Henrick, 2007 ▸).

## 3. Results and discussion

### 3.1. Gene identification, expression and purification

Genome sequencing of phage Tth15-6 resulted in one contig of about 76 kb. Analysis of the nucleotide sequence revealed a close similarity (95.2% identity with 70% query coverage) to the *Thermus* bacteriophage TSP4 isolated from hot springs in Tengchong, People’s Republic of China (Lin *et al.*, 2010 ▸). In addition, the phages G20c and P23-45 from the Geyser Valley in Iceland and P74-26 from the Uzon Valley in Kamchatka, Russia that infect *T. thermophilus* HB8 (Minakhin *et al.*, 2008 ▸) are related, although these homologous proteins have diverged significantly in amino-acid sequence from their counterparts from phages Tth15-6 and TSP4.

ORFs within the phage Tth15-6 genome were predicted by the *RAST* server and suggested the presence of a gene encoding a putative DNA-resolving enzyme/helicase-like enzyme of 155 amino-acid residues extending from coordinate nucleotide 18521 to nucleotide 18988 in the phage Tth15-6 genome. A sequence-similarity search with *BlastP* confirmed that the encoded protein, termed Hjc_15-6, showed sequence similarity to other deposited sequences of putative Hj-resolving enzymes.

**Figure 1:**
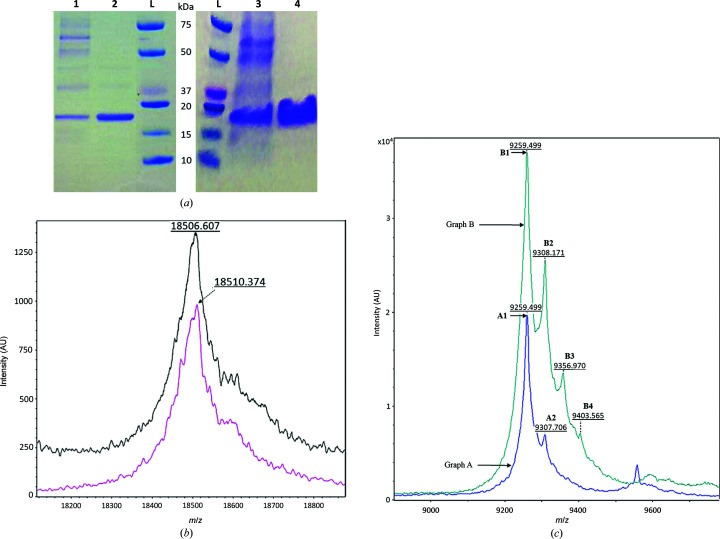
(*a*) Lanes 1 and 2: SDS–PAGE of crude extract supernatant and purified Hjc_15-6 protein produced in shake flasks using complex (LB) medium. Lanes 3 and 4 correspond to lanes 1 and 2 where the crude extract supernatant and purified Hjc_15-6 protein were instead produced in a fermenter using defined (mAT) medium. Lanes L contain molecular-mass markers (Bio-Rad). (*b*) Internally calibrated mass spectrogram (*z* = +1) of recombinant Hjc_15-6 protein overproduced in shake flasks using complex (LB) medium (upper black spectrogram) and Hjc_15-6 protein overproduced in a fermenter using defined (mAT) medium (lower pink spectrogram). (*c*) Externally calibrated mass spectrogram (*z* = +2) of overproduced Hjc_15-6 protein. The lower blue spectrogram (graph A) corresponds to Hjc_15-6 that was overproduced in shake flasks using complex (LB) medium and the upper green spectogram (graph B) corresponds to Hjc_15-6 protein that was overproduced in a fermenter using defined (mAT) medium. Peaks A2 and B2 have an additional mass of 96.4 and 97.3 Da compared with peaks A1 and B1, respectively, and peaks B3 and B4 have an additional mass of 97.6 and 93.2 Da compared with peaks B2 and B3, respectively.

The gene encoding the putative Hj-resolving enzyme from phage Tth15-6 was subsequently PCR-amplified and cloned in frame with a C-terminal hexahistidine tag in a pET-series expression vector; after heat-shock transformation it was expressed in *E. coli* BL21(DE3) cells using either 1 l complex (LB) medium in shake flasks or 2.5 l defined (mAT) medium in a fermenter. The yield was comparatively high using both cultivation strategies, and the crude cell extract constituted almost 50% of the soluble target protein according to qualitative estimation after visualization with SDS–PAGE (Fig. 1 ▸ *a*). Typically, a total of approximately 75 mg of Hjc_15-6 was obtained after protein purification by nickel-affinity chromatography using both cultivation strategies. The production of selenium-derivatized Hjc_15-6 using 0.5 l defined (mAT) medium supplemented with SeMet resulted in a total of approximately 0.8 mg protein after nickel-affinity purification.

### 3.2. Molecular mass, pI and oligomeric state

The deduced amino-acid sequence of full-length Hjc_15-6 (155 amino acids) corresponds to a theoretical average molecular mass of 17 580 Da (without the hexahistidine tag), which increases to 18 646 Da when the C-terminal hexahistidine tag (LEHHHHHH) is included. The molecular mass of purified recombinant Hjc_15-6 in solution was successfully determined by MS. The purified Hjc_15-6 included the hexahistidine tag, although the first amino acid (Met) of the full-length protein was lost, most likely due to N-terminal methionine excision (NME) after overproduction (Bonissone *et al.*, 2013 ▸). With these modifications, the recombinant protein has an expected theoretical average molecular mass of 18 514 Da and a theoretical monoisotopic mass of 18 502 Da. The calibrated MS spectrum confirmed that the actual molecular mass of recombinant Hjc_15-6 is between these values. Furthermore, molecular masses of 18 507 Da for recombinant protein produced in complex (LB) medium and 18 510 Da for protein produced in defined (mAT) medium were determined by MS, showing that no misincorporation of amino acids occurred in either medium (Fig. 1 ▸ *b*).

Interestingly, MS analysis revealed a difference in the additional groups interacting with Hjc_15-6 from the two production strategies. The MS spectra of Hjc_15-6 expressed in defined (mAT) medium had three additional peaks with higher molecular masses, suggesting that some of the protein had one or two additional molecular groups with estimated molecular masses of 97.3 and 97.6 Da and also a third group with a calculated molecular mass of 93.2 Da (peaks B2, B3 and B4 in Fig. 1 ▸ *c*). The corresponding MS spectra of Hjc_15-6 expressed in complex (LB) medium had only one additional peak, indicating a molecular group with an estimated molecular mass of 96.4 Da (peak A2 in Fig. 1 ▸ *c*). These groups may be either sulfate (molecular mass 96 Da) or phosphate (molecular mass 95 Da), as discussed further below. The pI value of Hjc-15_6 was estimated as 9.7.

Dynamic light-scattering (DLS) measurements demonstrated that the polydispersity was only 9 ± 1%, confirming the monodispersity of the pure Hjc_15-6 sample regardless of the production medium and/or the cultivation mode [complex (LB) medium and shake flasks or defined (mAT) medium and fermenter]. Furthermore, the radius of 3.5 ± 0.5 nm in both cases indicates that the oligomeric state of the resolvase is either a dimer or a dimer of dimers. Analysis of the polydispersity of the coordinates in the SeMet-derivatized structure (PDB entry 7bgs) and the native structure (PDB entry 7bnx) of Hjc_15-6 derived using *PISA* suggests that Hjc_15-6 is more likely to occur in a tetrameric form, with Δ*G* ^dis^ values of 6.1 and 8 kcal mol^−1^, respectively, for a tetrameric assembly and −1.7 and −1.3 kcal mol^−1^, respectively, for a dimeric assembly. However, since Hj-resolving enzymes are reported to use only two active sites in the dimeric state (Wyatt & West, 2014 ▸), it may be that Hjc_15-6 adopts a tetrameric form for purposes other than its Hj-resolving activity. Due to this uncertainty, Hjc_15-6 is discussed and displayed below both as a homodimer and a dimer of homodimers.

### 3.3. Sequence analysis

**Figure 2:**
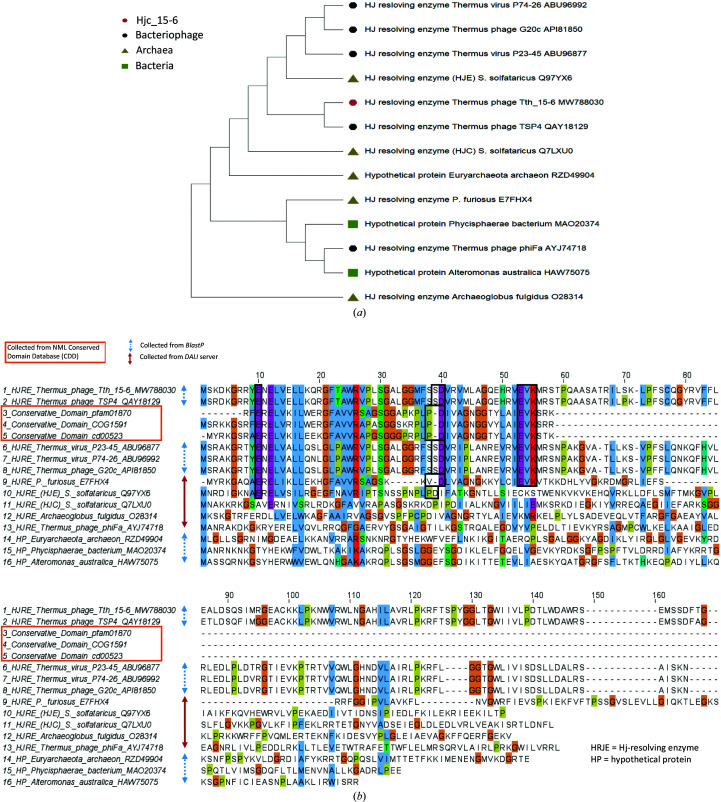
(*a*) Phylogenetic tree made in *MEGA X* based on proteins found in *BlastP* with a query coverage of 45% or more (obtained in September 2021) along with four structurally related enzymes found by the *DALI* server. (*b*) All protein sequences in the phylogenetic tree are displayed along with three relevant conservative domains found in the NML Conserved Domain Database (CDD). Rows 1–9 correspond to Hjc_15-6 aligned with the Hj-resolving enzyme from *P. furiosus* (found by the *DALI* server) and all phage proteins found in *BlastP*, except for *Thermus* phage phiFA. The alignment was performed in *Jalview* (Waterhouse *et al.*, 2009 ▸) using the *ClustalO* web service with default settings and aligned output order, with colouring by *ClustalX*. In this study, the conserved residue Glu10 is suggested to complement the PD-(D/E)*X*K catalytic motif and the respective SD-EVK motif (found in the phage sequences except for that of *Thermus* phage phiFA) to build a proposed signature. The proposed signature is marked with black boxes and consists of residues E^10^-SD^40^-EVK^55^ in Hjc_15-6 and other aligned Hj-resolving enzymes originating from phages (rows 1–2 and rows 6–8) and residues E^10^-PD^40^-EVK^55^ in the conservative domains (rows 3–5).

A search with *BlastP* shows that the amino-acid sequence of Hjc_15-6 has the highest sequence similarity to putative Hj-resolving enzymes from other thermophilic phages that infect *Thermus* species, including *Thermus* phage TSP4 (GenBank QAY18129.1; 96.1% identity, 100% query coverage), *Thermus* phage P74-26 (GenBank ABU96992; Minakhin *et al.*, 2008 ▸; 51.9% identity, 98% query coverage), *Thermus* virus P23-45 (GenBank ABU96877; Minakhin *et al.*, 2008 ▸; 52.6% identity, 98% query coverage) and *Thermus* phage G20c (GenBank API81850; Xu *et al.*, 2017 ▸; 51.3% identity, 98% query coverage). Their total alignment score is 99 ± 1% and the *E*-values are 3.0 × 10^−35^ or less. The putative Hj-resolving enzyme from the thermophilic phage *Thermus* phage phiFa (GenBank AYJ74718.1) has a relatively low sequence similarity to Hjc_15-6, despite having quite a high query coverage (29.4% identity, 87% query coverage). In the phylogenetic tree (Fig. 2 ▸ *a*) all proteins with a sequence query cover of 45% or more are displayed, along with four structurally related enzymes of archaeal origin. In Fig. 2 ▸(*b*) the amino-acid sequences of the proteins in the phylogenetic tree are displayed along with three additional relevant conservative domains found in the NML Conserved Domain Database (CDD). Hjc_15-6, which is displayed in the top row in Fig. 2 ▸(*b*), is aligned with the sequences in rows 2–9, which include the conservative domains and Hj-resolving enzymes from *P. furiosus* and all phage proteins found in *BlastP* except for *Thermus* phage phiFA.

Sequence analysis of Hjc_15-6 indicates that approximately the first 60 amino acids (the N-terminus) of the 155-residue sequence represent the more conserved part of the enzyme. In this part, a search in *InterPro* shows homology with two superfamilies: the tRNA endonuclease-like domain superfamily (IPR011856) and the restriction endonuclease type II-like superfamily (IPR011335). A search in the NLM CDD (version 3.18, 55570 PSSMs, default settings and full mode) generated a match with the restriction endonuclease-like superfamily (cd01037) and the three conserved domains COG1591, Archeal_HJR (cd00523) and Hjc (pfam1870) (mentioned above and displayed in Fig. 2 ▸ *b*), which are all described as Hj-resolving enzymes of archaeal type. The Hjc domain is known to be the archaeal equivalent of RuvC. However, it has quite a different amino-acid sequence (Komori *et al.*, 2000 ▸), and the Archeal_HJR domain has been described to show structural similarity to type II restriction endonucleases.

According to *InterPro*, the tRNA_endonuclease-like domain superfamily (IPR011856) has overlapping entries with the three domain types tRNA intron endonuclease-catalytic domain-like (IPR006677), VRR-NUC domain (IPR014883) and PD(D/E)*X*K endonuclease (IPR021671). It has also been described to represent a structural domain found in three groups of endonucleases: TsnA endonucleases (Ronning *et al.*, 2004 ▸; Xu *et al.*, 2017 ▸), tRNA-intron endonucleases (Li *et al.*, 1998 ▸) and Hjc-type resolving enzymes (classified as archaeal Holliday junction endonucleases; Nishino *et al.*, 2001 ▸; Xu *et al.*, 2017 ▸). The Restrct_endonuc-II-like superfamily (IPR011335) has been described to represent the core structure found in most type II restriction endonucleases.

Type II restriction endonucleases are involved in protecting bacteria and archaea against invading foreign DNA. Most of them are homodimeric or tetrameric enzymes with a similar structural core. They require Mg^2+^ ions for catalysis and cleave DNA independently of ATP at defined sites of 4–8 bp in length. Even if they differ in the details of the recognition process, it has been suggested that they evolved from a common ancestor (Xu *et al.*, 2017 ▸; Pingoud & Jeltsch, 2001 ▸; Pingoud *et al.*, 2005 ▸; Nakonieczna *et al.*, 2009 ▸).

A comparison of type II restriction endonucleases and other restriction endonucleases belonging to the PD-(D/E)*X*K superfamily illustrates that these enzymes have a similar core which harbours the active site (one per subunit) and comprise a five-stranded mixed β-sheet flanked by α-helices (Pingoud *et al.*, 2005 ▸; Venclovas *et al.*, 1994 ▸; Pingoud & Jeltsch, 2001 ▸; Nishino *et al.*, 2001 ▸; Winkler, 1992 ▸), which matches Hjc_15-6, as discussed below. Proteins belonging to the PD-(D/E)*X*K superfamily have various functions such as replication, restriction, DNA repair and tRNA–intron splicing (Steczkiewicz *et al.*, 2012 ▸). The PD-(D/E)*X*K motif is known to be a catalytic sequence motif that is involved in binding Mg^2+^ and in cleavage of the phosphodiester bond in the substrate DNA backbone (Dupureur & Dominguez, 2001 ▸; Pingoud & Jeltsch, 2001 ▸; Pingoud *et al.*, 2014 ▸). Based on extensive analysis, Pingoud and coworkers pointed out that a majority of the experimentally characterized type II restriction endonucleases for which full-length sequences are available belong to the PD-(D/E)*X*K phosphodiesterase superfamily, which also includes other nucleases; for instance *Sulfolobus solfataricus* Hj-resolving enzyme (Pingoud *et al.*, 2014 ▸).

To summarize, amino-acid sequence analysis with *InterPro* and CDD suggests that Hjc_15-6 may be classified as an archaeal type of Hj-resolving enzyme that shares common features with many other endonucleases, not least with the type II restriction endonucleases that belong to the PD-(D/E)*X*K superfamily.

In the alignment between Hjc_15-6 and the conservative domain sequences (Fig. 2 ▸ *b*), it is obvious that all conservative domains align with the PD-(D/E)*X*K motif. Hjc_15-6 residues 39–40 and 53–55 align rather well with this motif; however, the hydrophobic proline is now replaced by the polar residue serine (Fig. 2 ▸ *b*, row 1).

Several attempts have been made to improve the classification of the PD-(D/E)*X*K superfamily (Aravind *et al.*, 2000 ▸; Kosinski *et al.*, 2005 ▸; Laganeckas *et al.*, 2011 ▸; Steczkiewicz *et al.*, 2012 ▸). Daiyasu and coworkers investigated whether Asp40, Glu53 and Lys55 (in the alignment above) were critical for activity in the archeal Hj-resolving enzyme from *P. furiosus* (the residues are marked with black boxes in row 9 of the alignment in Fig. 2 ▸ *b*) by replacing them with alanines. The study showed that all of these residues were crucial for catalytic activity (Daiyasu *et al.*, 2000 ▸). Hence, the archaeal Hj-resolving enzyme from *P. furiosus* is related to the type II restriction endonucleases. However, as seen in the alignment, its catalytic motif is VD-(D/E)*X*K rather than PD-(D/E)*X*K (Fig. 2 ▸ *b*).

It has been suggested that archaeal Hj-resolving enzymes, together with Mrr (methylated adenine-recognition and restriction)-like endonucleases, should define a new family (Aravind *et al.*, 2000 ▸). This family has the endonuclease fold and the notable conservation of three motifs [which are part of the PD-(D/E)*X*K catalytic motif identified above] centred around a constellation of charged residues that could form the active site. These three motifs are described as follows: (G/P)*X*_(4)_E*X*_(9–11)_G(F/Y) (motif I), hDhh*X*p (motif II) and hhh(E/D/Q)hK (motif III), where *X* is any amino acid, p is any polar amino acid and h is a hydrophobic amino acid. Glu in motif I is strictly conserved (Aravind *et al.*, 2000 ▸) and may therefore be crucial for catalytic activity. The N-terminal motif (motif I) is predicted to form a helix, and the other two motifs form β-sheets. By comparing these results with other closely related families, it has been suggested that archaeal Hj-resolving enzymes coordinate a divalent cation (Mg^2+^) via Asp in motif II, Glu or Gln in motif III and one of the O atoms of the scissile phosphodiester group. Lys in motif III is likely to contact the phosphate of the DNA backbone (Aravind *et al.*, 2000 ▸).

The conclusions mentioned above align rather well with Hjc_15-6, giving the following three motifs: (G)*X*_(4)_E*X*_(10)_GF (motif I), pDhp*X*h (motif II) and pphEhK (motif III). The exceptions in Hjc_15-6 are that some surrounding residues are classified as polar in motifs II and III rather than hydrophobic. Despite this difference in hydrophobicity, it may be suggested that a putative catalytic site for Hjc_15-6 is Glu10–Asp40–Glu53–Lys55, where Asp40 takes part in binding an Mg^2+^ ion together with Glu53 and one of the O atoms of the scissile phosphodiester group, and Lys55 makes contact with the DNA backbone.

The close relationship between the sequences of Hjc_15-6 and putative Hj-resolving enzymes from thermophilic phages can be seen in rows 1–2 and 6–8 in the alignment in Fig. 2 ▸(*b*). Interestingly, except for the putative Hj-resolving enzyme from *Thermus* phage phiFa, all of the phage proteins display the three-part catalytic motif E-SD-EVK. Yet, at the same time, the corresponding motifs found in Hj-resolving enzymes from other species are E-PD-EVK or even E-VD-EVK, as in the case of the archeal Hj-resolving enzyme from *P. furiosus*. Based on this observation and the discussion above, we propose that Hjc_15-6 and several other Hj-resolving enzymes originating from thermophilic phages will have a three-part signature motif corresponding to E-SD-EVK. Furthermore, based on previous studies, as discussed above, it is suggested that Glu from motif I, Asp from motif II and Glu and Lys from motif III are the crucial catalytic site residues responsible for phosphodiester bond cleavage in conjunction with Mg^2+^ on the substrate DNA.

### 3.4. Crystal structure

The crystal structure of Hjc_15-6 was determined to 2.54 Å resolution using SAD with selenium-derivatized protein (Table 2 ▸). Eight Se atoms were found in the asymmetric unit with an occupancy of >25%, and two Hjc_15-6 molecules. After solvent flattening the mean figure of merit (FOM) was 0.59, and after automatic model building *R*_work_ and *R*_free_ were 0.33 and 0.34, respectively. After further model building and refinement, the final model included two Hjc_15-6 molecules, polypeptide chains *A* and *B*, with residues 5–28, 39–85, 98 and 100–149 of chain *A* and residues 5–29, 39–90, 98 and 100–150 of chain *B* visible in the electron density as well as eight sulfate or phosphate ions (modelled as sulfates) and 37 water molecules (PDB entry 7bgs). This model was then used to determine the native structure of the Hjc_15-6 protein. The native structure is similar to the SeMet-derivatized structure and includes polypeptide chains *A* and *B*, with residues 5–28, 39–85, 98 and 100–149 of chain *A* and residues 5–28, 38–90, 98 and 100–150 of chain *B* visible in the electron density as well as eight sulfate or phosphate ions (modelled as sulfates) and 32 water molecules (PDB entry 7bnx).

### 3.5. Structural analysis

**Figure 3:**
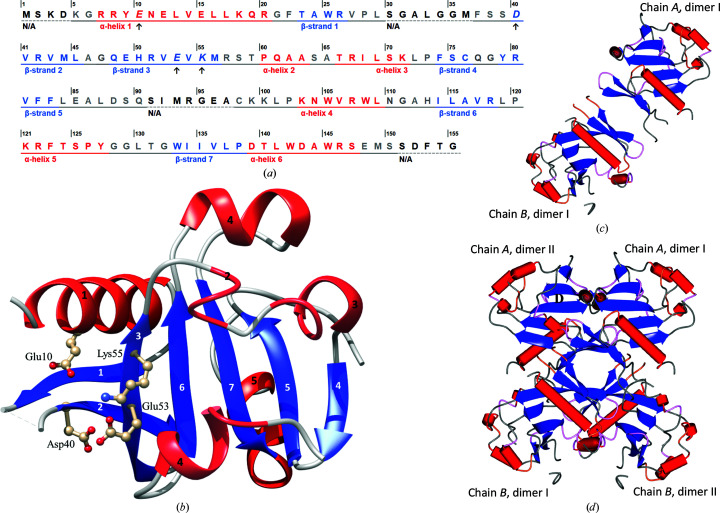
(*a*) The secondary structure of the Hjc_15-6 monomer is displayed based on data from both chains *A* and *B* as described in *UCSF Chimera* and *CCP*4*MG*. (*b*) Hjc_15-6 protein monomer coloured by secondary structure with α-helices (red helices) and β-sheets (blue arrows) numbered in order from the N-terminus to the C-terminus. The image also includes the putative active site with the catalytic residues Glu10, Asp40, Glu53 and Lys55 labelled and presented in ball-and-stick representation (Fig. 3*b* was produced in *UCSF Chimera* with the native coordinates of PDB entry 7bnx chain *B*). (*c*) Hjc_15-6 protein displayed as a homodimer. Chains *A* and *B* are slightly differently modelled. (*d*) Hjc_15-6 protein displayed as a dimer of homodimers. In (*c*) and (*d*), Hjc_15-6 is also coloured by secondary structure, with α-helices as red tubes and β-sheets as blue arrows; both images were produced in *CCP*4*MG*.

**Figure 4:**
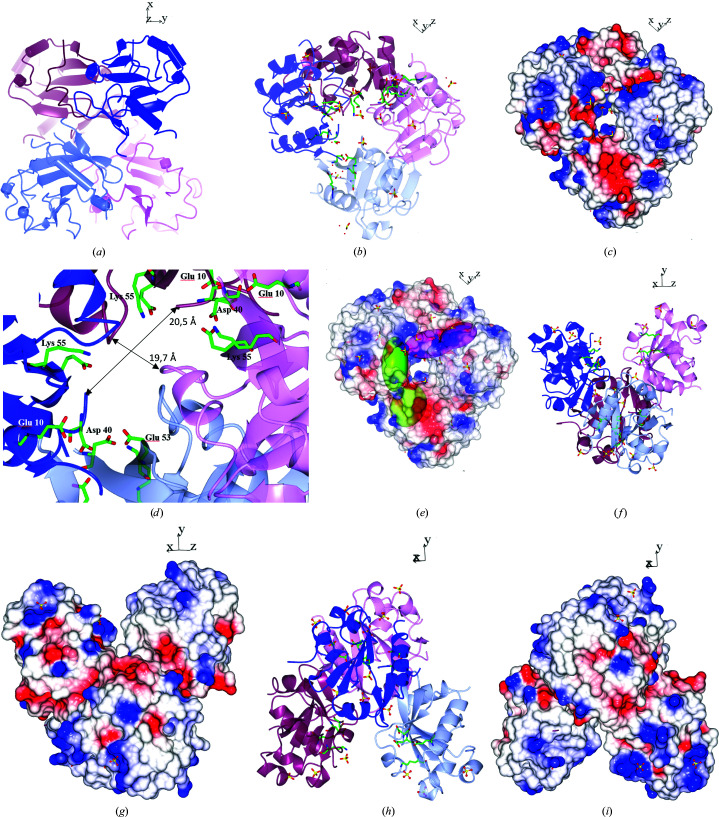
Images of Hjc_15-6 as a tetramer. Chains *A* and *B* in dimer I are shown as dark and light blue ribbons and chains *A* and *B* in dimer II are shown as dark red and pink ribbons in (*a*), (*b*), (*d*), (*f*) and (*h*). The active-site residues Glu10, Asp40, Glu53 and Lys55 are drawn as cylinders and coloured according to atom type in (*b*), (*d*) and (*g*). Phosphate/sulfate groups are drawn as sulfate groups with cylinders coloured according to atom type in (*b*), (*c*), (*e*), (*f*) and (*g*). In (*e*) the electrostatic surface potential is transparent, with bloboids of active-site residues coloured in green (dimer I) and pink (dimer II). In (*a*) it can be seen that dimer I is in front of dimer II. In (*b*)–(*e*) all images are displayed at the same angle, revealing a tunnel in the centre of the Hjc_15-6 tetramer that is viewed as a close-up in (*d*), where two distances have been measured. (*f*) and (*g*) present views rotated from the bottom to the top by 90° from those in (*b*)–(*e*), revealing a cleft between chain *A* in dimer I and chain *B* in dimer II. In (*h*) and (*i*) the tetramer is further rotated 90° to the left compared with (*f*) and (*g*), revealing another cleft between chain *A* in dimer II and chain *B* in dimer I.

The secondary structure of the Hjc_15-6 monomer is composed of seven β-strands and six α-helices (Figs. 3 ▸ *a* and 3 ▸ *b*). Hj-resolving enzymes have so far been reported to function as dimers (Wyatt & West, 2014 ▸); however, both the DLS and *PISA* results in this study suggest that Hjc_15-6 may occur as a dimer of homodimers. Hjc_15-6 is presented as a homodimer in Fig. 3 ▸(*c*) and as a dimer of homodimers in Fig. 3 ▸(*d*), both coloured according to secondary structure. It can be seen in Fig. 3 ▸(*d*) that the Hjc_15-6 tetramer has a X-shaped appearance, in which the two dimers seem to cross each other at the centre of the tetramer with an angle of 90°. However, in Fig. 4 ▸(*a*) it can be seen that dimer I (dark and light blue) is in the front of dimer II (dark red and pink).

As discussed previously, the putative active-site residues in the proposed motif E^10^-SD^39–40^-EVK^53–55^, except for residue 39, agree with archaeal Hj-resolving enzymes, as predicted by others (Aravind *et al.*, 2000 ▸). Moreover, this signature is situated within the most conservative part of Hjc_15-6, where the putative active-site residue Glu10 (motif I) is located in α-helix 1, Asp40 (motif II) in β-sheet 2 and Glu53 and Lys55 (motif III) in β-sheet 3 (Fig. 3 ▸ *b*). However, Ser39 prior to Asp40 (motif II) is not shown to be part of β-sheet 2. This may be due to the gap between residues 30 and 37, which are not seen in any chain in the two models. It can be seen in Figs. 3 ▸(*a*) and 3 ▸(*b*) that the first 60 residues (N-terminus) of Hjc_15-6, which represent the conservative part of the protein, and the core around the signature motif have the typical endonuclease fold built by an αβββαβ topology (Kinch *et al.*, 2005 ▸).

The electron-density map of Hjc_15-6 suggested that each monomer has two bound sulfate or phosphate groups with full occupancy. In addition, there are two further sulfate- or phosphate-binding sites in both monomers that have an occupancy of 0.5 and/or have high *B* values. Nishino and coworkers suggested that sulfate groups in the *P. furiosus* Hjc enzyme act as scaffolds for the N-terminal conformation. They also suggested that the sulfate ions are essential for successful crystallization (Nishino *et al.*, 2001 ▸). The latter observation was also experienced in this study when crystallizing Hjc_15-6. The fully occupied sites are displayed in Fig. 4 ▸(*b*), along with the proposed active-site residues Glu10, Asp40, Glu53 and Lys55 in the suggested catalytic motif E^10^-SD^40^-EVK^55^ of the Hjc_15-6 protein, viewed as a tetramer (a dimer of homodimers). The image is drawn at an angle where the two dimers are situated alongside each other. It is interesting to note that at this angle it can be seen that the tetramer has a tunnel through its centre. Fig. 4 ▸(*c*) shows the surface electron density of the tetramer displayed at the same angle as in Fig. 4 ▸(*b*). In the close-up of the tunnel (Fig. 4 ▸ *d*), several of the active-site residues from chain *A* in both dimer I and dimer II are oriented close to the surface of the tunnel. The overall orientation of the active-site residues is displayed in Fig. 4 ▸(*e*), where the electrostatic surface potential is shown along with the active-site residues represented as bloboids. The distances between the closest residues across the tunnel are estimated to be around 20 Å, as calculated using *CCP*4*MG* and displayed in Fig. 4 ▸(*d*). It might be that DNA binds in this cavity. However, this is uncertain for several reasons. Firstly, as discussed above, we are not convinced that Hjc_15-6 is a tetramer. Secondly, the tunnel may be smaller than modelled since some residues are missing. Finally, the Hj-resolving activity should be covered by the first third of the conservative part of the Hjc_15-6 protein. There are also nonconservative residues, for instance Gly111, on the tunnel surface (with a distance of 19.7 Å between Gly111 in chains *A* and *B* in dimer II). However, it cannot be excluded that DNA may bind in the tunnel, and if this is the case it might be that Hjc_15-6 has an additional function beyond acting as an Hj-resolving enzyme.

Suppose that the Hjc_15-6 tetramer is rotated 90° from the bottom to the top from the view displayed in Figs. 4 ▸(*b*)–4 ▸(*e*). In this case, we can see a cleft on the top of the tetramer between chain *A* in dimer I and chain *B* in dimer II (Fig. 4 ▸ *h*). If the tetramer is rotated one more time by 90° to the left, it can also be seen that there is another cleft between chain *A* in dimer II and chain *B* in dimer I, where the DNA substrate backbone may also bind (Fig. 4 ▸ *i*).

A search for structural alignment using the *DALI* server generated 11 unique PDB entries that matched Hjc_15-6 (chain *A*) with a *Z*-score of >4, a %id PDB of ≥20 and a Lali (length of alignment) of ≥73. The PDB entries corresponded to four unique UniProt entries that are all thermophilic archaeal Hj-resolving enzymes. Two of the Hj-resolving enzymes, Q97YX6 (HJE_SACS2) and Q7LXU0 (HJC_SACS2), have a significantly different substrate specificity from each other, cleaving Holliday junctions either in a sequence-independent (HJE_SACS2) or a sequence-dependent manner, even though both proteins originate from genes in *Saccharo­lobus solfataricus* (Kvaratskhelia & White, 2000 ▸). The other two Hj-resolving enzymes originate from *Archaeoglobus fulgidus* and *P. furiosus* (see Table 3 ▸ for an overview of the PDB entries and the corresponding proteins).

**Figure 5:**
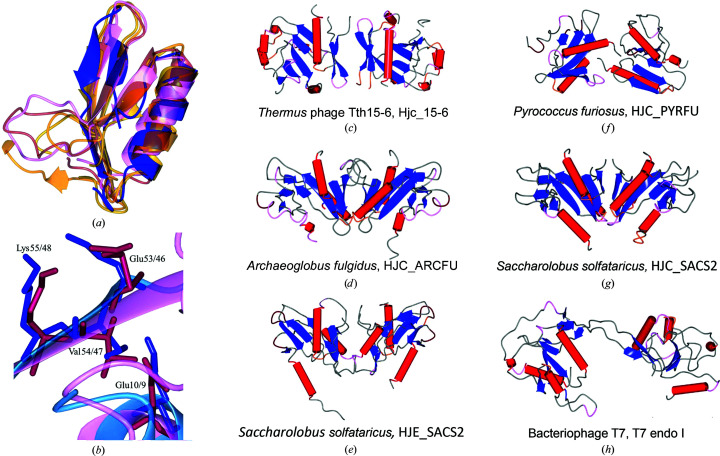
(*a*) The four best-matching monomers (listed in Table 3 ▸) of archaeal Hj-resolving enzymes found in *DALI* are superimposed on the Hjc_15-6 monomer (blue). Only the conservative parts of the monomers up to amino-acid residue 60 are displayed. (*b*) Residues 1–60 (chain *B*, PDB entry 1ipi) of the *P. furiosus* enzyme (pink and dark red) are superimposed on residues 5–60 of Hjc_15-6 (light and dark blue). The image illustrates how well the catalytic motif Glu10 and Glu53-Val54-Lys55 of Hjc_15-6 matches the corresponding residues in the *P. furiosus* enzyme. (*c*)–(*h*) The dimer organizations of Hjc_15-6, the four best-matching archaeal Hj-resolving enzymes and phage T7 endonuclease I are displayed. Colouring is according to secondary structure (α-helices as red tubes and β-sheets as blue arrows). All images in Fig. 5 ▸ were produced in *CCP*4*MG*.

All archaeal Hj-resolving enzymes from the best structurally matching molecules (as obtained in *DALI*) were superposed on the Hjc_15-6 monomer (chain *A*), and are displayed up to residue 60 in Fig. 5 ▸(*a*). It can be seen that all enzymes correspond very well to each other in this conservative part. When superposing only the 60-residue conservative part in *CCP*4*MG*, it was found that Hjc from *P. furiosus* matches Hjc_15-6 best. The structural orientation of Glu10, Glu53, Val54 and Lys55 from the catalytic motif of Hjc_15-6 is strikingly similar to the corresponding residues from Hjc of *P. furiosus* as seen in Fig. 5(*b*) ▸. However, all archaeal Hj-resolving enzymes give an r.m.s.d. (root-mean-square deviation) value between 1.74 and 1.88 Å. Even if the conservative parts of Hjc_15-6 and the archaeal Hj-resolving enzymes are very similar to each other, it can be seen that their dimeric organizations are somewhat divergent (Figs. 5*c*–5*g* ▸), except for HJC_ARCFU (*A. fulgidus*) and HJC_SACS2 (*S. solfataricus*), which are quite similar in their dimeric organizations (Figs. 5*d* and 5*g* ▸). Based on the observations above, we may conclude that Hjc_15-6 has a typical archaeal endonuclease fold and a dimer organization that resembles those of other archaeal Hj-resolving enzymes.

To date, there are (to the best of our knowledge) three available structures of Hj-resolving enzymes originating from phages. One of them is a mutant variant from phage bIL67 that resembles the *E. coli* RuvC fold (Green *et al.*, 2013 ▸) and the other two are (and have the folds of) T4 endonuclease VII and T7 endo I, respectively (Bond *et al.*, 2001 ▸). Hjc_15-6 should be most closely related to T7 endo I, since the active site of T7 endo I is much more similar to archaeal Hjc-resolving enzymes than to T4 endonuclease VII (which is almost totally α-helical) and the RuvC fold (which has a different topology; Hadden *et al.*, 2001 ▸). Yet, T7 endo I did not come up as a result in the *DALI* search. Furthermore, their dimeric organizations are quite different from each other, as can be seen in Figs. 5 ▸(*c*) and 5 ▸(*h*). The difference between Hjc_15-6 and T7 endo I shows that this study presents a new fourth fold for Hj-resolving enzymes originating from phages. This aligns well with the previous report that both T7 endo I and archaeal Hj-resolving enzymes belong to the endonuclease fold, although T7 endo I is quite divergent within this class (Aravind *et al.*, 2000 ▸).

### 3.6. Activity screening for resolving enzyme activity

**Figure 6:**
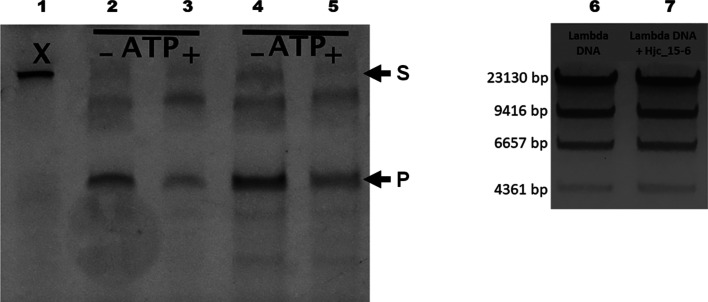
Cleavage of a DNA molecule in the form of a Holliday junction by the Hjc_15-6 enzyme. Lane 1 contains intact X-shaped dsDNA. The resolving enzyme activity of Hjc_15-6 is visualized in lanes 2–5 after incubation for 30 min at 50°C. The reaction mixtures in lanes 2–5 contained 10 pmol X-­shaped substrate DNA, 10 m*M* Tris–HCl pH 8.5, 100 m*M* KCl, 10 m*M* MgCl_2_ and 35 pmol purified Hjc_15-6 expressed in complex (LB) medium. Lanes 3 and 5 contained an additional 5 m*M* ATP, whereas the samples in lanes 2 and 4 were not treated with ATP. In lanes 4 and 5, the Hjc_15-6 used had been purified by dual chromatography to remove contaminants. In lane 6, 0.1 µg double-stranded blunt-end λ/HindIII DNA fragments were used as a control. In lane 7, a reaction mixture with 1 µg blunt-end λ/HindIII and 1.3 µg (approximately 35 pmol) Hjc_15-6 (purified twice) was applied after incubation for 30 min at 37°C. Except for the substrate DNA (1 µg blunt-end λ/HindIII) the reaction mixture is the same as in lane 4, indicating that Hjc_15-6 has no nuclease activity towards the nonbranched blunt-end dsDNA. S and P indicate the positions of substrate and product, respectively.

Activity screening demonstrated that purified recombinant Hjc_15-6 acts as a resolvase, cleaving Holliday-junction oligomers. Two affinity-chromatography techniques were exploited for Hjc_15-6 purification as a precaution against contamination when recombinant Hjc_15-6 was prepared for activity characterization. The reactions were performed with Hjc_15-6 in the presence of Mg^2+^, using marked fluorescent DNA shaped as Holliday junctions, with or without ATP. After 30 min of incubation at 50°C, the Hjc_15-6 digestion of DNA was visualized by TBE–PAGE as fragmented DNA that has migrated down the gel (Fig. 6 ▸ *a*). The cleavage reaction seems to be independent of the presence of ATP, which is a feature that Hjc_15-6 shares with type II restriction endonucleases (Pingoud & Jeltsch, 2001 ▸). Since no endonuclease activity was observed on polymerase-treated blunt-end λ dsDNA (Fig. 6 ▸ *b*) the activity screening demonstrates that the Hjc_15-6 sample is not contaminated with other endonucleases and that Hjc_15-6 has specificity towards Holliday junctions in contrast to nonbranched dsDNA. However, further studies are necessary for detailed characterization of the resolving activity and specificity of Hjc_15-6.

## 4. Conclusion

This study presents the first structure of an Hj-resolving enzyme, that of Hjc_15-6, originating from a *T. thermophilus* phage. Based on both sequence conservation and structural architecture, it can be assigned to the archaeal Holliday junction-resolving enzymes. The structure was initially solved using selenium-derivatized Hjc_15-6 (PDB entry 7bgs) and subsequently also with native Hjc_15-6 (PDB entry 7bnx). The enzyme demonstrates typical features of type II restriction endo­nucleases, in particular the endonuclease fold. Activity screening confirmed the Hj-resolving activity of Hjc_15-6; it does not cleave blunt-end λ/HindIII DNA fragments.

Sequence analysis and comparison with other publically available sequences of putative Hj-resolving enzymes from thermophilic phages and related studies have led us to propose that most Hj-resolving enzymes originating from thermophilic phages will have a three-part signature motif corresponding to E–SD–EVK and that the Glu (motif I), Asp (motif II) and Glu and Lys (motif III) residues are crucial residues responsible for phosphodiester bond cleavage in conjunction with Mg^2+^ on the substrate DNA. Furthermore, since Hjc_15-6 has an archaeal fold, this study demonstrates a new fourth fold for Hj-resolving enzymes originating from phages.

## Supplementary Material

PDB reference: Hjc_15-6, native, 7bnx

PDB reference: selenomethionine derivative, 7bgs

## Figures and Tables

**Table 1:** DNA oligomers used to build X-shaped DNA

Oligo 1	5′-CGAGTCGTTCGCAATACGGCTGTACGTATGGTCTCG-3′
Oligo 2	5′-CGAGACCATACGTACAGCACCGCTATTCATCGGTCG-3′
Oligo 3	5′-CGACCGATGAATAGCGGTCAGATCCGTACCTACTCG-3′
Oligo 4	5′-CGAGTAGGTACGGATCTGCGTATTGCGAACGACTCG-3′

**Table 2:** X-ray diffraction data-collection, structure-determination and refinement statistics for Hjc_15-6 Values in parentheses are for the highest resolution shell.

Protein	SeMet Hjc_15-6	Native Hjc_15-6
PDB code	7bgs	7bnx
Data collection†		
Space group	*C*222_1_	*C*222_1_
*a*, *b*, *c* (Å)	97.58, 103.71, 84.85	96.71, 104.17, 84.86
α, β, γ (°)	90, 90, 90	90, 90, 90
Resolution (Å)	30–2.50 (2.60–2.50)	29–2.55 (2.66–2.55)
*R*_merge_	0.111 (1.735)	0.067 (0.698)
*R*_p.i.m._	0.027 (0.560)	0.009 (0.186)
CC_1/2_	0.999 (0.631)	1.000 (0.953)
〈*I*/σ(*I*)〉	18.2 (1.1)	47.2 (2.9)
Completeness (%)	99.9 (100.0)	99.8 (98.7)
Multiplicity	16.7 (10.3)	50.9 (12.7)
No. of Se sites	8	
FOM (*CRANK*2)	0.59	
Refinement		
No. of unique reflections	15236	14269
*R*_work_/*R*_free_ ‡	0.236/0.258	0.230/0.254
No. of atoms		
Protein chains *A*/*B*	961/1025	952/1006
Sulfates/water	40/37	40/32
*B* factors (Å^2^)		
Protein chains *A*/*B*	91.9/95.2	90.9/89.5
Sulfates/water	100.0/67.9	98.8/66.2
R.m.s. deviations		
Bond lengths (Å)	0.009	0.009
Bond angles (°)	2.0	0.97
Ramachandran statistics (%)		
Favoured	97.0	96.6
Allowed	3.0	2.6
Disallowed	0.0	0.4
*MolProbity* score	1.68 [99th percentile]	1.80 [99th percentile]
Clashscore	5.29 [99th percentile]	5.15 [99th percentile]

† Two crystals were used for the SeMet Hjc_15-6 data and one crystal was used for the native data.

‡ 5% of the data were used for the *R*_free_ set.

**Table 3:** All PDB entries that match Hjc_15-6 (chain *A*) with a *Z*-score of >4, a %id PDB of ≥20 and a Lali (length of alignment) of ≥73 using the *DALI* server All corresponding proteins are Hj-resolving enzymes that originate from archaeal and thermophilic species. R.m.s.d. values are given from the lowest to the highest value noted for all compared chains of each species, and the PDB code of the best matching molecule is indicated in bold.

PDB entry	Protein name	UniProtKB entry	Protein length	R.m.s.d. (Å)	Species
**2wiz (chain *B*)**/2wcz/2wcw/2wj0/2wiw	Holliday-junction resolving enzyme Hjc	O28314 (HJC_ARCFU)	139	2.8–3.1	*Archaeoglobus fulgidus*
**1ob8 (chain *B*)**	Holliday-junction resolving enzyme Hje	Q97YX6 (HJE_SACS2)	135	3.0–3.1	*Saccharolobus solfataricus*
**1ipi (chain *B*)**/1gef	Holliday-junction resolving enzyme Hjc	E7FHX4 (HJC_PYRFU)	123	3.0–3.1	*Pyrococcus furiosus*
**1ob9 (chain *A*)**/4tkd/1hh1	Holliday-junction resolving enzyme Hjc	Q7LXU0 (HJC_SACS2)	143	3.0–4.1	*Saccharolobus solfataricus*
